# First Unmanned Aerial Vehicle Observation of Epimeletic Behavior in Indo-Pacific Humpback Dolphins

**DOI:** 10.3390/ani12111463

**Published:** 2022-06-05

**Authors:** Tabris Yik-To Chung, Heysen Hei-Nam Ho, Henry Chun-Lok Tsui, Brian Chin-Wing Kot

**Affiliations:** 1 Centre for Applied One Health Research and Policy Advice, City University of Hong Kong, 83 Tat Chee Avenue, Kowloon, Hong Kong, China; ytchung@cityu.edu.hk (T.Y.-T.C.); cltsui9@cityu.edu.hk (H.C.-L.T.); 2 Department of Infectious Diseases and Public Health, Jockey Club College of Veterinary Medicine and Life Sciences, City University of Hong Kong, 83 Tat Chee Avenue, Kowloon, Hong Kong, China; heinamho@cityu.edu.hk; 3 Southern Marine Science and Engineering Guangdong Laboratory (Guangzhou), Guangzhou 511458, China

**Keywords:** altruism, biotelemetry, cetacean, drone, kinship

## Abstract

**Simple Summary:**

In this study, we reported a case of epimeletic behavior in Indo-Pacific humpback dolphin. Using a combination of drone and conventional photography, we were able to comprehensively document individual body condition, swimming pattern, and group behavior during the epimeletic event. Our research highlighted the application of drones in wildlife research to provide important insights into the health, behavior, and ecology of free-ranging populations.

**Abstract:**

Epimeletic behavior has been reported in various species of cetaceans and sometimes in wild populations during vessel-based surveys. Epimeletic behavior in cetaceans involves complex social interactions which have been described using observational and acoustic studies. The recent advances in unmanned aerial vehicle (UAV) technology allowed its application in wildlife research and frequently in cetaceans in conjunction with vessel-based surveys. This article is the first report of intraspecific epimeletic behavior of Indo-Pacific humpback dolphins (*Sousa chinensis*) in Hong Kong waters using a combination of UAV- and vessel-based photography. Using both techniques, we were able to observe and qualitative analyze the individual body condition, group behavior, and swimming pattern during the epimeletic event. This study highlighted that UAVs can be used to observe the complex social behaviors and interactions of cetaceans from the aerial angle while keeping a minimal level of disturbance to the animals. Aerial footage can also be quantitatively analyzed to provide further insights on the group behaviors of cetaceans. The application allows efficient assessment of health, behavior, and ecology of wild animals, offering valuable opportunities for researchers working on free-ranging populations.

## 1. Introduction

The Indo-Pacific humpback dolphin (*Sousa chinensis*) resides in the coastal waters of Southeast Asia and the Eastern Indian Ocean, which is listed as vulnerable in the IUCN Red List [1]. The largest *S. chinensis* population locates in the Pearl River Estuary region, including the subpopulation in Hong Kong waters, and the size of this population has been rapidly declining under various anthropogenic influences [2,3,4,5,6]. Several incidents of epimeletic behavior of *S. chinensis* have been reported using vessel-based observations, mostly as nurturant behavior towards dead calves [7,8,9]. Cheng and his team [10] reported a case of epimeletic behavior on a dead *S. chinensis* calf with onboard observation and acoustic monitoring in Guangxi, China. Increased length and complexity of vocalization were documented during the epimeletic event in comparison with social context [10]. An incident of interspecific epimeletic behavior between *S. chinensis* and the sympatric Indo-Pacific finless porpoise (*Neophacaena phocaenoides*) has also been reported [11]. Epimeletic behavior is not uncommon among cetaceans and typically involves one or more adults supporting a dead calf for a prolonged period [12]. During an epimeletic event, the focal adult may intend to resuscitate the incapacitated newborn without acknowledging their death [13]. The strong social bond of cetaceans may contribute to delayed abandonment and more frequent observations of epimeletic behavior [14]. Due to the relatively low energetic cost of carrying a small-sized neonate on water and their lack of olfactory and gustatory senses [15], epimeletic behavior of cetaceans can last for weeks [14]. In cetaceans, the focal adult often demonstrated protective behavior when observers attempted to separate the carcass, and in some cases of successful removal, the focal adult may resort to carrying an inanimate object as a surrogate to prolong the epimeletic behavior [12,16]. The focal adult–offspring pair is often accompanied by other conspecifics in the same social group, which may exhibit diverse behaviors, including but not limited to standing-by, supporting, socializing, or even aggressive behavior [10,17,18,19]. Acoustic records associated with epimeletic behavior of the highly vocal *Tursiops truncatus* have been reported where the nurturant adults emitted distress calls to seek aid from conspecifics [18,20]. 

However, it is challenging to distinguish the behavior of each individual by either vessel-based observation or acoustic monitoring [10,21]. For example, the horizontal angle of vessel-based observation limits the ability to observe and trace individuals through behavior events, especially in larger groups, while acoustic monitoring may reflect vocalization at a group level instead of a specific individual [10]. Unmanned aerial vehicle (UAV) technology is a modern approach used to assess and monitor the abundance and distribution of different megafauna [22]. Advancement in UAV technology also offers new opportunities for studying the health and behavior of cetaceans remotely and non-invasively from an aerial angle [23,24,25,26]. This article reports the first comprehensive observation of the intraspecific epimeletic behavior of *S. chinensis* in Hong Kong waters using UAV.

## 2. Materials and Methods

Vessel surveys on *S. chinensis* were routinely conducted in the South and West Lantau waters of Hong Kong for health assessment of the local cetacean populations. Groups of *S. chinensis* in the area were located by vessel-based observers using binoculars, followed by the documentation of their health status and behavior using photography and UAV, which were supplemented with written visual observations. Digital single-lens reflex cameras with 400 mm image-stabilized lenses were used to take photographs in a horizontal view from the vessel. A UAV (Phantom 4 Pro, DJI, Shenzhen, China) was used to record aerial footage at an angle of depression between 60° and 90°. The UAV was manually launched on the deck of the vessel and maintained at 15 m above sea level. This altitude was chosen as UAV deployment at this altitude caused no disturbance (as indicated by immediate behavior changes) to *S. chinensis* in our trial flights and as documented in other small cetaceans (*T. truncatus* and *Delphinus delphis* [27]). Restricted by the battery (PH4-5870, DJI, Shenzhen, China), the UAV used has a maximum flight time of 30 min, but the duration of each flight was limited to 20 min to account for uncertainties in weather and retrieval. All photographs and aerial footages were time-stamped and synchronized to allow simultaneous analysis of the biological and behavioral characteristics of *S. chinensis* from different angles.

## 3. Results

A group of *S. chinensis* was encountered between 12:24 and 13:52 on 12 June 2020, in South Lantau waters between Fan Lau Tung Wan and Tai Long Wan (22.198706 N, 113.878790 E). Four separated aerial footages, lasting for 20 min in total, were recorded and analyzed along with vessel-based photographs and visual observations by experienced dolphin observers. When the group was spotted, there were seven purse seiners in the area. Some individuals were intermittently interacting with and foraging alongside the adjacent fishing activities. No signs of disturbance or distress by the research vessel or the UAV were noted from the animals throughout the sighting.

**Figure 1:**
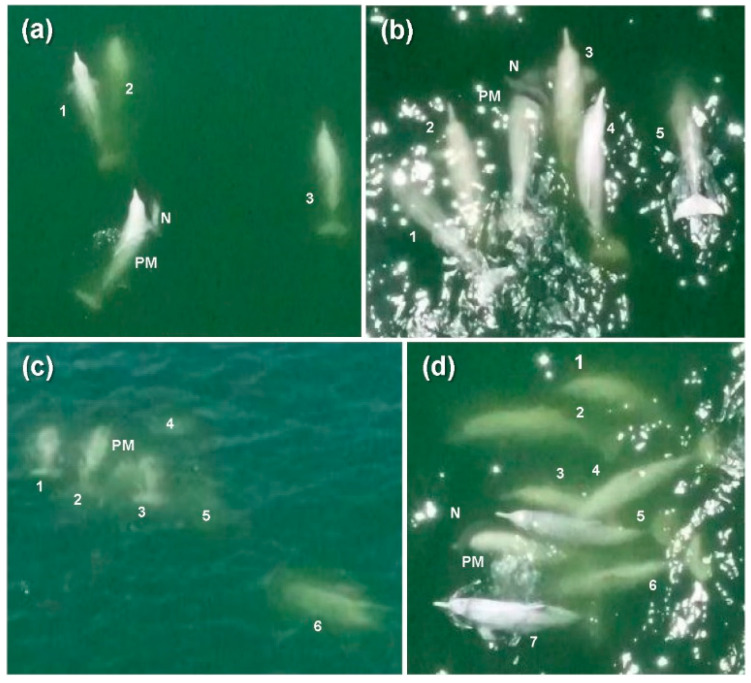
Aerial photographs of the group of Indo-Pacific humpback dolphins *Sousa chinensis* extracted from the aerial footage. Photographs were extracted at (**a**) 12:55; (**b**) 13:17; (**c**) 13:25; and (**d**) 13:31 to show the gradually increasing number of escorting conspecifics alongside the neonate (N) and presumed mother (PM).

**Figure 2:**
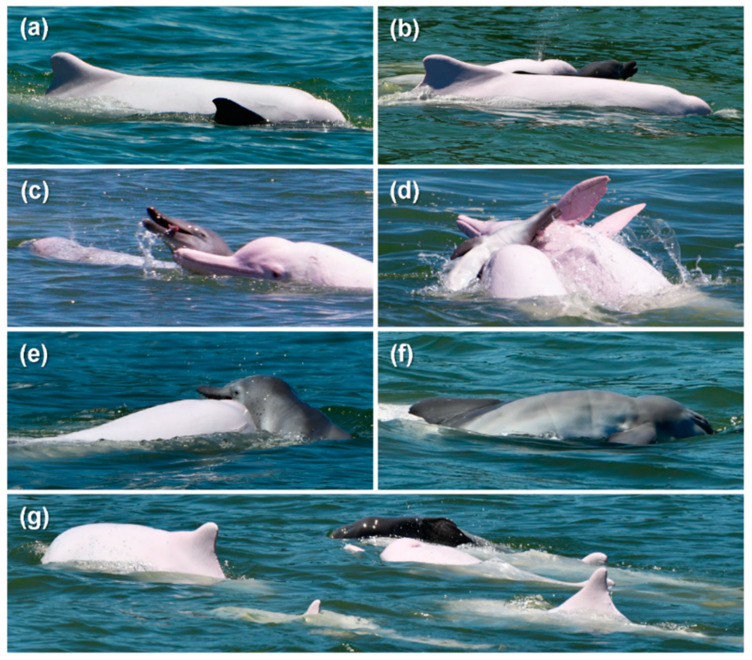
Vessel-based photographs of the group of Indo-Pacific humpback dolphins *Sousa chinensis* taken from the research vessel. (**a**) The neonate positioned adjacent to the presumed mother in an echelon position; (**b**) the neonate supported by the presumed mother and an escort; (**c**–**e**) the neonate in a flaccid state with tilted head and protruding tongue lifted by the presumed mother and escorts; (**f**) the presence of fetal folds on the neonate; (**g**) multiple conspecifics escorting the pair (neonate and presumed mother) in a synchronous raft-like formation.

From the aerial footage, the neonate was initially sighted alongside four adult conspecifics in the vicinity which were swimming in the same direction (Figure 1a). The neonate was first seen in a natural prone orientation, which was verified by vessel-based photographs taken around the same time (Figure 2a). One of the adults, designated as the presumed mother (PM) due to the constant proximity and interaction with the neonate throughout the observation, was supporting the neonate on the right side of the body with the pectoral flipper in an apparent echelon position [28] (Figure 1a and Figure 2a). Subsequently, the neonate sank, while all four of the adults turned and circled underwater in different directions (video footage at https://youtu.be/0e-JzucRMdo, accessed on 28 May 2022). After approximately 20 s, all four adults realigned and swam in the same direction. The neonate was then seen again supported on the right side of the PM, while the other adults travelled in proximity as escorts (Figure 2b). It was uncertain whether the neonate was debilitated or deceased at first, but over the aerial observation, it became obvious that the neonate was deceased as it was seen to be unresponsive when being supported by the rostrum of the PM (Figure 2b,d). This was also supported by photographic evidence throughout the sighting as the neonate appeared to be in a flaccid state with tilted head and protruding tongue (Figure 2c–e). Fetal folds displayed on the body when lifted also confirmed its very young age (Figure 2f). The lift-and-sink events continued throughout the sighting (video footage at https://youtu.be/rYW925Pg08s, accessed 28 May 2022), which is typical of epimeletic behavior [20,29].

Throughout the aerial footage, up to eight individuals, including the PM, were observed in the group. The PM was assisted by an increasing number of escorts over time, with three at initial sighting (12:55; Figure 1a), to five (13:17; Figure 1b), six (13:25; Figure 1c), and up to seven (13:31; Figure 1d). The gradual increase in group size over the observation may be the result of natural socialization or recruitment by the PM using vocalization [10]. These escorts were swimming parallel to the PM–neonate pair in a raft-like formation [18] (Figure 1b–d and Figure 2b,g). The synchronous presence of multiple conspecifics to defend deceased young have been reported in odontocetes and other highly sociable mammals as a result of kin selection or altruism [29,30]. Besides being supported by the pectoral flipper and rostrum of the PM as a common maternal behavior [31], the neonate was also occasionally seen pushed by the escorts using the dorsum or pectoral flippers (Figure 2c,d). Cetaceans have been reported to lift newborns to the sea surface to facilitate breathing or movement [18,32]. From our photographic evidence, no noticeable rake marks or external injuries were seen on the neonate (Figure 2d–f), and no display of aggressive or coercive behaviors such as rapid chasing, ramming, or tossing as described by a previous study on the infanticidal behavior in *S. chinensis* [33] was seen among the escorts. The entire group, including the PM, swam calmly with no sudden changes in direction throughout the sighting, except when the neonate sank and the group circled underwater, presumptively to retrieve the neonate. After over 90 min of focal observation, the PM–neonate pair still appeared to be tightly associated. The sighting was ended and the research vessel departed without disturbance to the animals. The neonate was not retrieved nor found later for postmortem examination and the cause of death was undetermined.

## 4. Discussion

During the vessel survey, experienced observers noted at least six dolphins (Figure 2g), whereas the review of the UAV footages amended the total number of adults in the group to eight (Figure 1d). The discrepancy was due to the non-synchronized surfacing of the animals as observed from the vessel, while the aerial angle allowed visualization of the entire group in shallow water. UAV allows clear tracking of individual animals. Swimming patterns, as well as socializing and foraging behaviors were more visible from the aerial view. Each of the individuals can be traced throughout the footages, which was difficult in conventional vessel-based observation. Aerial footage can be quantitatively analyzed to provide insights into the group behavior of cetaceans. For instance, individual or group swimming patterns can be quantified (e.g., direction, rate of reorientation, surface rate). Aerial footage can also be used to observe unique foraging behavior in cetaceans [34]. On the other hand, vessel-based photography from a horizontal angle allows documentation of biological signs and health conditions [35], as well as individual identification. In our case, the individual that exhibited constant contact with the neonate (i.e., the PM) was observed with a scar situated right laterally to its blowhole (Figure 2a,e), thus allowing it to be distinguished from other individuals in the group (i.e., the escorts). The combination of vessel-based and aerial observation is critical for unveiling the kinship of each individual and the dynamics among each member. Acoustic recording of the event, if included, may provide a further understanding of their behaviors [10,18,20].

The reaction to anthropogenic disturbance is dependent on the nature of the animals and is often species specific [27,36]. Both visual stimuli and acoustic footprint of UAVs may potentially disrupt the behaviors of cetaceans [37,38]. *S. chinensis* in Hong Kong frequently interact with humans. They are known to feed near purse seiners [39] and may swim near dolphin watching boats and research vessels (Kot B.C.W., pers. obs.). In our previous trials, no behavioral changes (e.g., swimming depth, surfacing frequencies, reorientation, interruption of feeding) were noted when the UAV was operated at 15 m above sea level. No apparent responses to disturbance (e.g., side float, spy hop) nor stress-induced behaviors (e.g., pectoral slap, tail lob) were induced unless the altitude was reduced to <10 m. Thus, the altitude of 15 m was maintained as our standard flight protocol, with behavior of the animals cautiously monitored to minimize potential disturbance. Throughout this sighting, no signs of disturbance or avoidance were observed, with the group travelling without stress-induced behavior or sudden reorientation, except when the neonate sank and the group circled underwater to retrieve the neonate.

## 5. Conclusions

UAVs offer an alternative angle for cetacean surveys which can advance existing research. On top of photogrammetric studies on body conditions [40,41], behaviors [42,43,44], and distributions [45,46], UAVs were also deployed to collect cetacean blows for the investigation of respiratory microbiomes [47,48]. One of the constraints of UAV is the battery life of the aircraft, which limits each flight to under 30 min and unavoidably interrupts the duration of each observation or sampling. Adverse weather such as strong wind may hinder the deployment of UAV. Lighting conditions may also influence the quality of the footage. For instance, this epimeletic event was observed at noon when the sun was casting tremendous glare on the sea surface, which reduced the clarity of the footage shot perpendicularly. Technical adaptations such as polarized light filters may reduce light reflection and will be considered in future work. This study is the first to document epimeletic behavior of *S. chinensis* in Hong Kong waters using UAV. The application can transcend some of the limitations of conventional vessel-based observations in cetacean behavioral and ecological assessments.

## Data Availability

The video footages presented in this study are openly available on YouTube at https://youtu.be/0e-JzucRMdo and https://youtu.be/rYW925Pg08s, accessed on 28 May 2022.
